# Detection of *KIT* Mutations in Systemic Mastocytosis: How, When, and Why

**DOI:** 10.3390/ijms252010885

**Published:** 2024-10-10

**Authors:** Daniela Cilloni, Beatrice Maffeo, Arianna Savi, Alice Costanza Danzero, Valentina Bonuomo, Carmen Fava

**Affiliations:** Department of Clinical and Biological Sciences, University of Turin, Mauriziano Hospital, 10128 Turin, Italy; beatrice.maffeo@unito.it (B.M.); arianna.savi@gmail.com (A.S.); alicecostanza.danzero@unito.it (A.C.D.); valentina.bonuomo@unito.it (V.B.); carmen.fava@unito.it (C.F.)

**Keywords:** *KIT*, gene mutations, systemic mastocytosis, digital PCR

## Abstract

More than 90% of patients affected by mastocytosis are characterized by a somatic point mutation of *KIT*, which induces ligand-independent activation of the receptor and downstream signal triggering, ultimately leading to mast cell accumulation and survival. The most frequent mutation is *KIT* p.D816V, but other rarer mutations can also be found. These mutations often have a very low variant allele frequency (VAF), well below the sensitivity of common next-generation sequencing (NGS) methods used in routine diagnostic panels. Highly sensitive methods are developing for detecting mutations. This review summarizes the current indications on the recommended methods and on how to manage and interpret molecular data for the diagnosis and follow-up of patients with mastocytosis.

## 1. Introduction

Mastocytosis is a rare clonal disease characterized by an accumulation of mast cells in various organs, including the skin, bone, gastrointestinal tract, lymph nodes, and spleen [1,2]. It can affect any age, with a male-to-female ratio of about 1:1. According to the Orphanet platform (https://www.orpha.net), the prevalence of mastocytosis in the adult population is about 1 case per 10,000 subjects. There are no prevalence data for the pediatric forms. Systemic mastocytosis almost always arises in adulthood, generally between the ages of 20 and 50. In children, the onset of mastocytosis occurs in 75–80% of cases within the first two years of life. Most cases are sporadic, but rare familial forms have been described [1,2,3]. From a molecular point of view, mastocytosis is mostly associated with a somatic point mutation in the gene encoding the KIT receptor for stem cell factor (SCF). The most frequent *KIT* gene mutation is D816V, present in approximately 85–90% of adult cases and about one third of pediatric cases [4,5,6]. Mastocytosis is associated with a wide variety of clinical manifestations, generally due to the inappropriate release of mediators by mast cells. Among these the most frequent are itching, urticaria, angioedema, flushing, nausea, vomiting, abdominal pain, diarrhea, anaphylaxis, cardiovascular symptoms like syncope or presyncope, tachycardia, osteopenia, or osteoporosis. Additional symptoms are psychological and neurological manifestations such as headaches, syncopal spells, seizures, transient chorea, vertigo, paraesthesia, and chronic inflammatory demyelinating polyneuropathy [7]. In addition, the presence of skin involvement is frequent with diffuse red-brown lesions, especially on the trunk and limbs with the characteristic Darier’s sign when rubbed [8]. In aggressive forms, mastocytosis also presents signs of organ dysfunction related to mast cell infiltration, including hypersplenism, pathological fractures, ascites, malabsorption, and cytopenias. The rarity of this condition and the multiplicity of clinical manifestations necessitate the integration of various disciplines for accurate diagnosis and appropriate therapeutic management [8].

About 10% of systemic mastocytosis is associated with another hematologic neoplasm, primarily chronic myelomonocytic leukemia (CMML), chronic myeloproliferative neoplasms (MPN), and myelodysplastic syndromes (MDS) [9,10].

The WHO classification 2022 defines precise criteria for the diagnosis of systemic mastocytosis [9]. The major criterion is based on the presence of multifocal dense infiltrates of mast cells with more than 15 mast cells aggregated in bone marrow (BM) and/or other extracutaneous organs. Minor criteria include (i) the presence of more than 25% of spindle-shaped or atypical mast cells in mast cell infiltrates detected in bone marrow or other extracutaneous organ histological sections, or more than 25% of mast cells with immature or atypical appearance in bone marrow smears. (ii) Presence of the Asp816Val mutation in the *KIT* gene or any other activating *KIT* mutation in bone marrow, peripheral blood, or other extracutaneous organs. (iii) Expression of CD25 and/or CD2 and/or CD30 in mast cells in the bone marrow, peripheral blood, or other extracutaneous organs. It is underlined that CD30 is a marker recently introduced as a minor diagnosis criterion. CD30 is expressed in neoplastic mast cells in a majority of patients with advanced systemic mastocytosis (85%), whereas in most patients with indolent systemic mastocytosis only a few if any mast cells stained positive for this marker. (iv) Persistent serum tryptase levels >20 ng/mL. This latter must be modified in cases affected by hereditary alpha tryptasemia, and it is not applicable in cases of SM-AHN in which the associated neoplasm is myeloid. WHO classification divides Systemic Mastocytosis into six different entities: bone marrow mastocytosis (BMM), indolent systemic mastocytosis (ISM), smoldering systemic mastocytosis (SSM), aggressive systemic mastocytosis (ASM), systemic mastocytosis with an associated hematological neoplasm (SM-AHN), and mast cell leukemia (MCL) (Table 1).

According to the WHO 2022 classification [9], the diagnosis of systemic mastocytosis (SM) is based on the integration of laboratory parameters (serum tryptase levels, blood chemistry tests), bone marrow (BM) morphological parameters (BM biopsy, BM blood smear), genetic parameters (presence of *KIT* mutations, cytogenetic alterations, or other markers of clonality), cytometric/immunohistochemical parameters (aberrant expression of membrane antigens), radiological and clinical parameters. In the case of patients with suspected SM but with normal or slightly elevated tryptase, highly sensitive techniques of molecular biology, such as allele-specific qPCR (ASOqPCR) and digital droplet PCR (ddPCR), are necessary [11]. The diagnostic process starts from clinical suspicion and varies according to the detected tryptase level [12]. If the tryptase level is >25 ng/mL, a complete bone marrow evaluation is immediately indicated, whereas if the level is <15 ng/mL, it is suggested to perform periodic monitoring over time. If the level is between 15 and 25 ng/mL, the decision to proceed with a bone marrow evaluation is based on additional important parameters included in the REMA score, such as male gender, absence of urticaria/angioedema, and episode of syncope. A REMA score higher than 2 indicates a high probability of clonal mast cell disorder [13], the presence of the *KIT* p.D816V mutation in peripheral blood (PB), or the presence of additional symptoms suggestive of the disease help in the clinical decision to continue with diagnostic investigations.

Symptoms can be variable in type, number, location and intensity; in some cases, they are very mild or even absent, while in others, they are severe or life-threatening. Symptoms due to mediator release are also variable and include cutaneous, allergic, gastrointestinal, neuropsychiatric, osteoarticular symptoms and many others [1,2].

Anaphylaxis is certainly the most severe clinical manifestation of ISM. Its frequency is reported between 22% and 49% in adults and between 6% and 9% in children [14,15]. The triggers are numerous, with hymenoptera stings being the most frequent cause, followed by reactions to foods and drugs [16]. Idiopathic anaphylaxis can account for up to 39% of anaphylactic cases in adults. As in the general population, cofactors, such as alcohol, physical exercise, and sudden temperature changes can play a significant role, being determinants in about 26% of anaphylactic reactions. A preferential association between hymenoptera venom allergy and mastocytosis is well established. The prevalence of hymenoptera venom allergy in the European adult population is about 3%, whereas it rises to 20–30% in patients with clonal mast cell disorders [14,15,16,17].

## 2. KIT/CD117

KIT/CD117 is a type III tyrosine kinase (TK) transmembrane receptor that binds stem cell factor [18]. The *KIT* gene, located on chromosome 4q12, consists of 21 exons and codes for a glycoprotein of 145 kD and 976 amino acids. The structure of the receptor is very similar to other TK receptors with an N-terminal extracellular domain that binds SCF and a C-terminal intracellular domain [19]. The two domains are linked by a transmembrane domain. The sequences linked to kinase activity are all within the intracellular domain, which can be divided into the juxtamembrane domain, with an inhibitory function of TK activity, and the TK domain. The extracellular domain (ECD) includes five immunoglobulin-like domains important for ligand binding and receptor dimerization [18,19,20].

There are several isoforms of KIT generated by alternative splicing, including two that arise from the presence or absence of four amino acids: glycine–asparagine-asparagine–lysine (GNNK). The two major isoforms of KIT are expressed in the juxta-membrane region of the extracellular domain. These isoforms are generally coexpressed, often with the GNNK- variant as the predominant transcript. Although both isoforms have the ability to bind SCF, the GNNK-negative form has faster receptor phosphorylation capability and, therefore, a more robust downstream signal. This isoform in mice has revealed oncogenic potential. In patients with mastocytosis, the increased expression of the GNNK- isoform has been shown to correlate with increased percentage of neoplastic mast cells. In vitro experiments of mast cell transfection revealed that GNNK- overexpression is associated with increased granule formation, histamine content, and cell growth [21,22,23]. A third isoform results from the loss of a serine residue in the kinase domain. Finally, a fourth isoform results from a truncated transcript that gives rise to a truncated protein lacking kinase activity [21,22].

*KIT* is expressed in various cell types [24]. The signal activated by the binding between the receptor and SCF is involved in many biological processes, primarily cell survival, proliferation, and migration. In normal bone marrow, *KIT* is expressed in hematopoietic stem cells and plays an important role in self-renewal and differentiation into various types of mature cells. *KIT* is gradually downregulated during differentiation and remains expressed only in mast cells, natural killer (NK) cells, and dendritic cells (DCs), suggesting its role in immunity and inflammation [25,26]. Finally, *KIT* is not only expressed in hematopoietic stem cells but also in the prostate, liver, and heart, indicating its role in the stem cells of these organs as well [27,28,29]. KIT has also been shown to be crucial in regulating oogenesis and spermatogenesis, thus playing a crucial role in fertility.

## 3. Downstream Signaling of KIT

**Figure 1:**
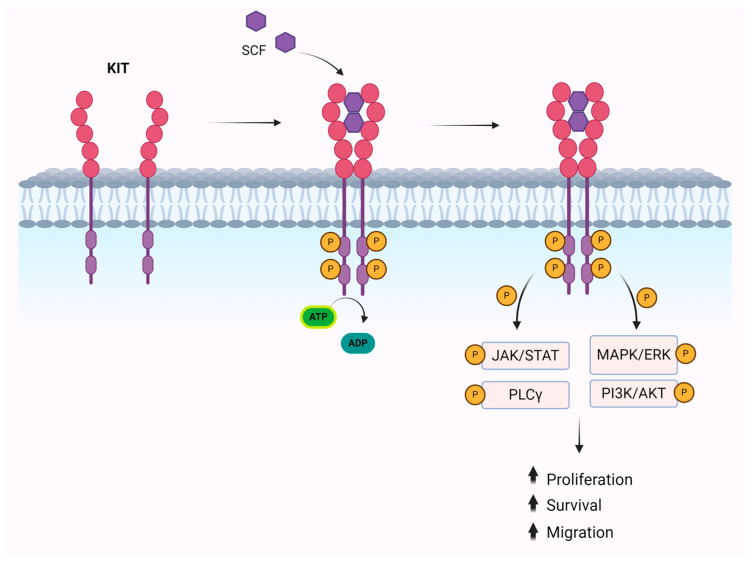
Illustration of KIT receptor activation by stem cell factor (SCF), triggering downstream signaling pathways: JAK/STAT, PLCγ MAPK/ERK, and PI3K/AKT, which collectively contribute to cell survival, proliferation, and migration.

The downstream signaling of KIT involves the activation of MAPK/ERK, PI3K/AKT, PLCγ, and JAK/STAT pathways [30]. The MAPK/ERK pathway is activated by the phosphorylation of Y703 and Y936 and plays a key role in transcription activation and cell proliferation [31]. The phosphorylation of Y721 activates the PI3K/AKT pathway, which promotes cell survival and escape from apoptosis [32]. In addition, the activation of the Src family of kinases (SFK) can occur, resulting in cell proliferation and survival through Akt phosphorylation and cell migration through the phosphorylation of focal adhesion kinase [31,32,33]. Finally, it has been shown that phosphorylation of some KIT tyrosine residues leads to JAK/STAT activation with the translocation of STAT proteins into the nucleus, where they target specific genes [32,33] (Figure 1). There are also negative feedback mechanisms to control the signal once the KIT receptor is activated. Among these, ubiquitination is the main mechanism. E3 ubiquitin-protein ligase c-CBL binds directly to the activated KIT receptor through Y568 and Y936 or indirectly through Grb2 to Y703 and Y936 or through the p85 subunit of PI3K [34,35]. Additional mechanisms include dephosphorylation and PKC-dependent serine phosphorylation. Finally, signal inactivation can occur through phosphatases such as Src homology region 2 domain-containing phosphatase 1, which causes KIT dephosphorylation.

## 4. KIT Is Activated in Many Tumors

The majority of KIT alterations found in cancer are associated with gain of function mutations leading to a constitutive activation of *KIT* independently from SCF. Gain-of-function mutations of *KIT* are detectable in many cancers, including gastrointestinal stromal tumor (GIST) [36,37], melanoma [38], seminoma [39], mastocytosis [40], and acute myeloid leukemia (AML) [41].

**Figure 2:**
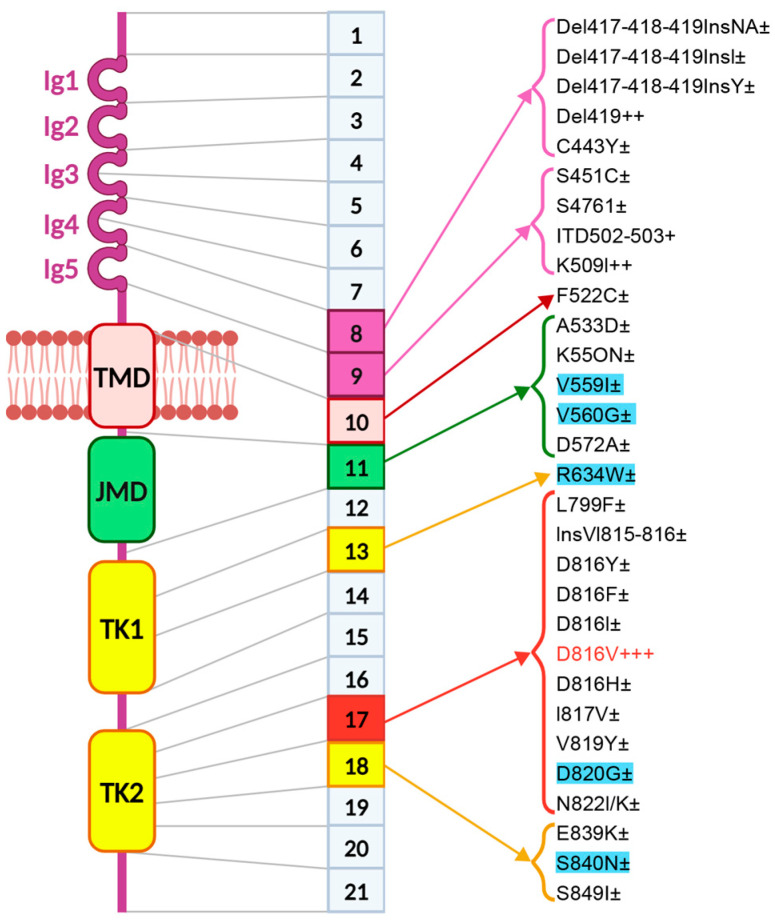
Entire spectrum of *KIT* mutations. On the left, the protein structure is shown with its various domains. In the center, the corresponding gene structure is depicted, highlighting the different exons that encode each domain. On the right, the mutations identified in the respective exons are indicated. Variant of uncertain significance (VUS) are highlighted in blue. TMD: trans-membrane domain. JMD: juxtamembrane domain. TK1: tyrosine kinase domain 1. TK2: tyrosine kinase domain 2. ± means that the mutation is present in less than 1% of the patients, + in 1%–5% of the patients, ++ in 5%–20% of the patients, +++ >20% of all adult patients. The figure is adapted from Valent P et al. [58].

More than 90% of mastocytosis cases are characterized by a somatic point mutation of *KIT*, the majority of which induces ligand-independent activation of the receptor and downstream signal activation, ultimately leading to mast cell proliferation and survival [42,43]. Different types of mutations have been described in mastocytosis [42,43,44]. A picture of *KIT* mutations is represented in Figure 2 and described in Table 2. Mutations located in the extracellular domain impact KIT dimerization, and mutations in the phosphotransferase domain impact on the kinase activity [45].

In children, *KIT* mutations often affect the extracellular domain of the receptor, while in adults the most frequent activating mutation is the *KIT* p.D816V found in the phosphotransferase domain encoded by exon 17 [46]. Other activating mutations, such as *KIT* V560G in the juxtamembrane domain and *KIT* D419del in the extracellular domain, are less common but found in patients with aggressive systemic mastocytosis (ASM), mast cell leukemia (MCL), and mast cell sarcoma [47,48,49]. Unlike adults, children with mastocytosis show the *KIT* p.D816V mutation in about 30% of skin biopsy cases, with other *KIT*-activating mutations in the extracellular domain present in about 40% of cases [46]. A recent study found no significant link between the progression (spontaneous regression or persistence into adolescence) and the type of *KIT* mutation in pediatric cases [50]. Finally, there are rare cases, not exceeding 5%, that do not present *KIT* mutations. Among these, we find almost all cases of mast cell sarcoma and cases with well-differentiated mast cells. However, there are also rare cases of classic systemic mastocytosis without *KIT* mutation [51]. Differential diagnosis should also consider the possibility of idiopathic mast cell activation syndrome (MCAS), defined by three diagnostic criteria: (i) recurrent mast cell-mediated symptoms affecting at least two organs without clonal mast cells proliferation or clear triggers; (ii) periodic elevations of MC mediator levels, especially tryptase levels; and (iii) improvement with treatments targeting mast cells. Additional mediators, including prostaglandin D2, histamine, and cysteinyl leukotrienes, are elevated. In this setting, mast cells are negative for CD2 and CD25 expression, and *KIT* is not mutated [52].

It is also interesting to note that studies of *KIT* mutations performed on cells other than mast cells show that about 30% of individuals with mastocytosis have a multilineage myeloid and/or lymphoid involvement of hematopoiesis [53,54,55,56]. Unlike what happens in the majority of patients where the *KIT* mutation is present only in the mast cell compartment, in these patients, the mutation is also found in basophils, eosinophils, neutrophils, as well as in B and T lymphocytes. This evidence suggests that the acquisition of the mutation occurs early in a multipotent hematopoietic progenitor and that the clonal origin of mastocytosis and the associated hematologic neoplasm is common. Multilineage involvement is more frequent in advanced systemic mastocytosis (AdvSM) or SM forms and less frequent in ISM forms, but when present in the latter, it is associated with a worse prognosis with a higher risk of disease progression [55]. This suggests that the earlier the mutation occurs hierarchically, the higher the risk of acquiring additional mutations and disease progression. In these patients, the *KIT* mutant allele frequency is a surrogate for the measurement of the multilineage involvement [57].

**Table 2:** Description of KIT mutations. Spectrum of mutations detected in different types of mastocytosis. Black colour indicates pathogenetic mutations. Green colour indicates mutations described either as pathogenetic or as variant of uncertain significance (VUS) by different sources. Pink colour indicated VUS. The reference sources are indicated in the right column.

Mutation	Pathogenetic/VUS	Type of Disease	Source
Del417-418-419Ins	Pathogenetic	Pediatric mastocytosis	[59]
Del419	Pathogenetic	Pediatric familial mastocytosis	[59]
C443Y c.1328G>A NM_000222.3	Pathogenetic	Pediatric mastocytosis	[59]
S451C c.1352C>G NM_000222.3	Pathogenetic	Systemic mastocytosis	[60]
S476I c.1427G>T NM_000222.3	Pathogenetic	Systemic mastocytosis	[59,61]
ITD502–503	Pathogenetic	Systemic mastocytosis	[59]
K509I c.1526A>T NM_000222.3	Pathogenetic	Systemic mastocytosis	http://clinvar.com
F522C c.1565T>G NM_000222.3	Pathogenetic	Systemic mastocytosis	[60]
A533D c.1598C>A NM_000222.3	Pathogenetic	Cutaneous mastocytosis	http://clinvar.com
K550N c.1650A>T c.1650A>C NM_000222.3	Pathogenetic	Systemic mastocytosis	http://clinvar.com
V559I c.1675G>A NM_000222.3	VUS		http://clinvar.com
V559I c.1675G>A NM_000222.3	Pathogenetic	Aggressive systemic mastocytosis	[59]
V560G c.1679T>G NM_000222.3	VUS		http://clinvar.com
V560G c.1679T>G NM_000222.3	Pathogenetic	Systemic mastocytosis	[62]
D572A c.1715A>C NM_000222.3	Pathogenetic	Systemic mastocytosis	[59]
R634W c.1900C>T NM_000222.3	VUS		http://clinvar.com
R634W c.1900C>T NM_000222.3	Pathogenetic	Familial mastocytosis	[59]
L799F c.2395C>T NM_000222.3	Pathogenetic	Systemic mastocytosis	[62]
InsVI815-816	Pathogenetic	Systemic mastocytosis	[54]
D816Y c.2446G>T NM_000222.3	Pathogenetic	Systemic mastocytosis	http://clinvar.com
D816F c.2446_2447delGAinsTT NM_000222.3	Pathogenetic	Pediatric mastocytosis	[60]
D816I c.2446_2447delGAinsAT NM_000222.3	Pathogenetic	Cutaneous pediatric mastocytosis	[63]
D816V c.2447A>T NM_000222.3	Pathogenetic	Systemic mastocytosis	http://clinvar.com
D816H c.2446G>C NM_000222.3	Pathogenetic	Systemic mastocytosis	http://clinvar.com
I817V c.2449A>G NM_000222.3	Pathogenetic	Systemic mastocytosis	[54]
V819Y c.2470_2472delGTGinsTAT NM_000222.3	Pathogenetic	Systemic mastocytosis	[64]
D820G c.2459A>G NM_000222.3	VUS		http://clinvar.com
D820G c.2459A>G NM_000222.3	Pathogenetic	Aggressive systemic mastocytosis	[59]
N822I c.2465A>T NM_000222.3	Pathogenetic	Systemic mastocytosis	http://clinvar.com
N822K c.2466T>A c.2466T>G NM_000222.3	Pathogenetic	Systemic mastocytosis	http://clinvar.com
E839K c.2515G>A NM_000222.3	Pathogenetic	Systemic mastocytosis	http://clinvar.com
S840N c.2519G>A NM_000222.3	VUS		http://clinvar.com
S849I c.2549G>T NM_000222.3	Pathogenetic	Systemic mastocytosis	[59]

## 5. Methods for the Detection of *KIT* Mutations

The frequency of the *-KIT* p.D816V mutation varies depending on the sensitivity of the method used to detect it, ranging from 30% to 95% of cases [65]. Although the presence of *KIT* mutations is a diagnostic criterion for systemic mastocytosis, there is currently no globally accepted standardized method for its detection.

Various non-quantitative (or semi-quantitative) methods have been employed in the past to detect the *-KIT* p.D816V mutation. These methods include RT-PCR combined with restriction fragment length polymorphism, nested RT-PCR followed by D-HPLC of PCR amplicons, and peptide nucleic acid-mediated PCR (PNA-PCR) [66]. Generally, these methods are known for their relatively low sensitivity and/or higher likelihood of false-negative results, particularly in cases with low mast cell burden and lack of multilineage involvement.

However, there is currently consensus that the search for *KIT* mutations should be performed using highly sensitive methods. The limit of detection (LOD) shall be less than or equal to 0.01% VAF [51].

This level of sensitivity is reached by ASO PCR or by droplet digital PCR (ddPCR) [67]. This recommendation stems from the fact that the allelic frequency of *KITp*.D816V can be very low because the mast cell component is often poorly represented in both PB and BM.

Kristensen and colleagues developed a highly sensitive method based on mutation-specific q-PCR for the detection of *KIT* mutation in mastocytosis [65]. This assay has a sensitivity ranging from 0.001% to 0.03% positive alleles and a specificity of 100%. They demonstrated for the first time that by using a highly sensitive and specific assay, it is possible to detect the *-KIT* mutation in blood in nearly all adult patients with mastocytosis [65]. Although qPCR is characterized by an adequate level of sensitivity, it also has the disadvantage that it requires a calibration material and there is no commonly accepted *KIT* calibrator. Currently, plasmid dilutions are used as a calibrator, but differences in the material used for calibration have made it difficult to standardize the method. To overcome this problem, attention has recently shifted to droplet digital PCR (ddPCR) [67].

Droplet digital PCR employs the distribution of diluted target nucleic acids across numerous reactions (partitions) to precisely quantify DNA molecules without the need for calibration material [68,69]. As a result, ddPCR has emerged as a new benchmark for quantifying mutant alleles at low variant allele fractions (VAF) [68].

Greiner and colleagues validated the ddPCR assay for *KIT* p.D816V [67]. They compared ddPCR to ASO PCR, demonstrating a high level of concordance between the two methods. Based on these data, the EU-US Cooperative Group considers both methods suitable for the quantification of *KIT* p.D816V allelic frequency from DNA [51].

Finally, although targeted ultradeep next-generation sequences have been developed for detecting *KIT* mutations [70], there are still limited data. It is important to underline that standard NGS techniques used for myeloid neoplasms workup have an insufficient limit of detection, usually ranging between 1% and 5% VAF, which is not adequate for *KIT* p.D816V detection in the majority of SM and particularly in patients with ISM.

## 6. Genomic DNA (gDNA) and mRNA: Which Is Better?

mRNA-based measurement of expressed allelic burden (EAB) is not interchangeable with DNA-based results. Recently, Naumann and colleagues [71], by analyzing a large number of samples, demonstrated that in patients with indolent systemic mastocytosis there is a high level of concordance between *KIT* p.D816V VAF and EAB, but in patients with AdvSM this correlation is weak. More in detail, in two-thirds of patients with AdvSM, this correlation is similar to ISM, while in one-third of the cases, *KIT* p.D816V EAB is at least 2-fold higher than VAF, suggesting an increased transcriptional activity of the mutant clone. The increased transcriptional activity is predictive for an advanced phenotype and a significantly inferior overall survival (OS). Although the mechanism that triggers the transcriptional activity remains to be explored, this activity probably plays an important role in the pathophysiology of systemic mastocytosis.

In 2016, Kristensen and colleagues [72] compared gDNA and mRNA in BM and PB. They found that depending on the expression level of *KIT* p.D816V, mRNA is potentially more sensitive than gDNA. However, gDNA-based assays are usually superior to mRNA in PB. The conclusion of this study is that mRNA-based analysis is the best tool to be used in BM, and gDNA-based assays are preferred over mRNA in PB. Needless to say, the absence of a complete concordance between gDNA and mRNA-based assays should be considered when interpreting the mutant allele frequency results.

## 7. What to Do When the Mutational Test Is Negative but the Clinical Suspicion Is Strong

In 5–10% of SM, the mutational analysis for *KIT* p.D816V results negative [51].

The EU-US Cooperative Group has considered these data and provided three scenarios that align with this possibility. The first is that the mutational load is below the detection limit, making it a false negative. The second is that it is truly negative. The third is that the patient may have a *KIT* mutation different from D816V that cannot be detected with the chosen method [51].

If the result was obtained with a method characterized by low sensitivity, the recommendation in these cases is to use a highly sensitive method to address scenario 1.

If the negative result was obtained on PB sample, it must be considered that a negative result on PB does not necessarily indicate that the patient is negative. The analysis should be repeated on a high-quality BM sample. It is described that mast cell enrichment by cell sorting can be required in selected cases to detect the presence of *KIT* mutation [53].

Furthermore, it is described that if an analysis on PB of gDNA results in borderline, typically the results are positive when reanalyzed using an increased amount of DNA. The quantity of DNA analyzed can therefore represent a limit.

If the result is negative in both BM and PB despite using a sensitive technique and the mast cell infiltrate is evident, then it is appropriate to look for other *KIT* mutations different from D816V. Whole-gene sequencing or PNA-PCR are recommended. In these cases, also the use of standard NGS, employed in many centers, can be useful, especially for assessment of VAF when other PCR-based methods are not available, while still considering the limited sensitivity of this method. Furthermore, NGS is frequently used when there is a clinical suspicion of other myeloid pathologies such as MDS and MPN, as described in paragraph 12.

## 8. What Material Should Be Used for the Mutational Analysis of *KIT*? Differences between BM or PB

For the diagnosis of systemic mastocytosis, different types of biological material can be used, commonly BM or PB but also tissue biopsies, including skin or gastrointestinal biopsies.

In cases of a reasonable suspicion of SM, the recommended material for diagnosis is BM aspirate collected in an EDTA tube, preferably fresh, although frozen material can also be used [65].

Although mature mast cells are generally not detected in PB, the use of PB for the analysis of *KIT* mutations is possible because an increased number of mast cell precursors are detectable in PB from SM patients. These circulating mast cell precursors usually carry *KIT* mutations, but the expression level of CD117 is low; therefore, low levels of mRNA are usually detectable in these cells [73]. In addition, a high VAF in PB generally is suggestive of a multilineage involvement.

## 9. How Frequent *KIT* p.D816V Allele Frequency Should Be Monitored?

The EU-US Cooperative Group believes that the frequency of evaluation depends on the type of disease, the stage, and the therapy. For example, for patients with ISM, the evaluation must be made at diagnosis and then is no longer considered necessary except in cases where progression is suspected. In AdvSM, especially in those under specific therapies, the VAF measurement should be repeated to monitor the treatment. VAF measurement must be repeated in all patients with SM who present signs of progression [51].

## 10. Significance of *KIT* p.D816V Allele Frequency

It is now clear that the *KIT* p.D816V mutant allele burden is a surrogate for the extent of multilineage involvement. In many patients with ISM, the mutation can be detected in many mature cells, including basophils, eosinophils, neutrophils, and B and T lymphocytes [53,54,55,56].

Many data confirm that multilineage involvement in ISM is one of the most important prognostic factors that impacts the probability of disease progression. Kristensen and colleagues reported a significant correlation between *KIT* p.D816V mutant allele frequency, serum tryptase levels, and the percentage of neoplastic mast cells [72]. These findings were confirmed by Erben and colleagues, who not only confirmed these data but demonstrated the correlation with the allele frequency and the type of disease and therefore with the overall survival [66]. Recently, Greiner and colleagues, by making use of ddPCR, established that *KIT* p.D816V mutant allele frequency correlates with MC infiltration, especially in tissues, and with serum tryptase levels. They also found that the allele frequency is higher in AdvSM compared to ISM and is an independent predictor of survival [73].

## 11. *KIT* p.D816V as a Marker of Response to Therapy

*KIT* p.D816V mutant allele frequency has been used as a biomarker to evaluate the response to treatment. The possibility of using VAF as a biomarker of response was described many years ago [74] following treatments with agents such as hydroxyurea, cladribine, and interferon alpha. In the same year, Erben suggested the use of VAF to monitor the disease after allogeneic transplant [66]. With the development of molecular therapies targeting KIT, the ability to monitor the response becomes critical. In this regard, emerging data confirm the utility of *KIT* p.D816V VAF as a biomarker. Specifically, the study by Jawhar and colleagues demonstrated that in patients treated with midostaurin, a 25% reduction in the expressed allelic frequency after 6 months of treatment is an independent prognostic factor for survival [75]. More recently, Reiter and colleagues analyzed the efficacy of avapritinib in previously treated patients with advanced systemic mastocytosis enrolled in the EXPLORER/PATHFINDER trials. They described a reduction in *KIT* p.D816V VAF in 66% of the cases, thus confirming the role of *KIT* as a biomarker [76].

## 12. Additional Molecular and Cytogenetic Lesions

In a subset of patients, mostly with advanced SM cases, additional mutations beyond *KIT* can be found [77,78,79,80,81]. These mutations are frequently associated with a worse prognosis. The additional mutations are mainly of two types. First, they can be mutations or translocations affecting driver genes associated with specific diseases, such as *JAK2* or *BCR::ABL,* and second, they can be mutations of genes that do not define a specific hematologic neoplasm, generally myeloid lineage associated mutations. Among these, for example, we find *CBL*, *KRAS*, *RUNX1*, *ASXL1*, *DNMT3*, *EZH2*, *TET2*, *SRSF2*, *SF3B1*, *U2AF1,* and *SETD2* [80]. Reference sequences are indicated in Supplementary Table S1. These mutations are frequently found in myeloid neoplasms, although they are not associated with a particular disease phenotype. The mutations of the first type, those associated with defined pathologies, are typical of mastocytosis associated with another hematologic neoplasm. For example, mastocytosis with *JAK2* mutation defines mastocytosis as associated with a chronic myeloproliferative disease or with chronic myelomonocytic leukemia. The mutations not associated with a specific subtype of hematologic neoplasm are often found in the advanced forms, mainly in forms associated with other hematologic diseases, and less frequently in mast cell leukemias.

Many studies have sought to give prognostic significance to these additional mutations. In particular, what is defined as the S/A/R panel, i.e., the presence and number of *SRSF2*/*ASXL1*/*RUNX1* mutations, represent a negative prognostic factor [81].

However, additional mutations do not always represent an unfavorable factor. Some studies published in the literature have clearly indicated that the presence of both *KIT* and *JAK2* mutations often characterizes a disease with two independent clones. In these patients, in the absence of S/A/R mutations, the 5-year overall survival rate is 77%, which is significantly different from that of patients with S/A/R mutations. In these patients, the allele frequency of both mutations, *KIT* and the additional one, also has clinical and prognostic significance. Indeed, a disease with minimal mast cell infiltration, a VAF of *KIT* p.D816V lower than 5%, and relatively low tryptase levels has significantly different clinical behavior compared to a disease with the same *KIT* allele frequency but many monocytes and a 50% VAF in one of the S/A/R genes [81].

Finally, some cytogenetic abnormalities detectable from karyotype examination have shown a prognostic role. In particular, abnormalities such as monosomy 7 and complex karyotype are associated with progression to very aggressive forms like acute mast cell leukemia. For this reason, cytogenetic investigation is strongly recommended in all advanced forms to evaluate clonal evolution and leukemic transformation [82,83].

## 13. Summary and Conclusions

Although there is still no standardization of methods to detect and measure *KIT* mutations, it is currently recommended to use highly sensitive techniques such as ASO-PCR and digital PCR.

Both BM and PB can be used for analysis, although the significance of allele frequency differs between the two materials.

A negative result in PB does not exclude the presence of a mutation in BM; if PB is negative, it is recommended to perform the analysis on BM as well.

The test can be conducted on either gDNA or RNA, with the precautions mentioned above.

*KIT* mutation can be used as a biomarker for therapy response.

NGS analysis with a myeloid panel to detect additional mutations is useful for establishing prognosis, as mutations in the SAR genes (SRSF2/ASXL1/RUNX1) are associated with a poorer prognosis.

## Figures and Tables

**Table 1:** WHO classification of systemic mastocytosis (2022). This table presents the World Health Organization (WHO) classification for systemic mastocytosis, outlining the various subtypes and diagnostic criteria. The criteria are based on clinical, histological, and genetic factors, which aid in the accurate diagnosis and management of the disease.

Bone Marrow Mastocytosis (BMM) *	SM Criteria Fullfilled, No Dense SM Infiltrates in Extramedullary Organs, Absence of B and C Findings, No Criteria for MCL or SM-AHN, No Skin Lesions.
Indolent Systemic Mastocytosis (ISM):	Bone marrow infiltration < 20% without associated hematologic disease, absence of C findings, and absence or presence of 1 B finding
Smoldering Systemic Mastocytosis (SSM):	Bone marrow infiltration < 20% without associated hematologic disease, absence of C findings, and presence of 2 or more B findings
Aggressive Systemic Mastocytosis (ASM):	Bone marrow infiltration < 20% without associated hematologic disease, presence of at least 1 C finding
Systemic Mastocytosis with Associated Hematologic Neoplasm (SM-AHN):	Mast cell percentage < 20% and bone marrow histology conclusive for hematologic disease (myelodysplastic syndrome or myeloproliferative syndrome).
Mast Cell Leukemia (MCL):	Patients with bone marrow infiltration >20% without signs of other hematologic diseases. Typically presents with C findings.such as anemia, thrombocytopenia, neutropenia, splenomegaly, hepatomegaly, malabsortion, weight loss, skeletal lesions.

* It is important to highlight that ddPCR performed on PB misses over 50% of BMM cases.

## Data Availability

Not applicable.

## Supplementary Materials

The following supporting information can be downloaded at: https://www.mdpi.com/article/10.3390/ijms252010885/s1.
