# Supplementary material for: Detection of KIT Mutations in Systemic Mastocytosis: How, When, and Why
Source: Int J Mol Sci. 2024 Oct 10;25(20):10885. doi: 10.3390/ijms252010885 (PMC11507058; doi:10.3390/ijms252010885)
Supplement: Supplementary file 1 [file ijms-25-10885-s001.zip › ijms-3207134-supplementary.pdf]

Supplementary Table S1

**RefSeq transcripts**
SRSF2: NM_001195427.2
ASXL1: NM_015338.6
RUNX1: NM_001754.5
CBL : NM_005188.4
KRAS: NM_004985.5
DNMT3A: NM_022552.5
EZH2: NM_004456.5
TET2: NM_001127208.3
SF3B1: NM_012433.4
U2AF1: NM_006758.3
SETD2: NM_014159.7
JAK2: NM_004972.4

Supplementary Table S1 Reference sequences of the genes that can be mutated in SM
